# The PCP pathway regulates Baz planar distribution in epithelial cells

**DOI:** 10.1038/srep33420

**Published:** 2016-09-14

**Authors:** Benoit Aigouy, André Le Bivic

**Affiliations:** 1 Aix Marseille Univ., CNRS, Institute of Developmental Biology of Marseille (IBDM), case 907, 13288 Marseille cedex 09, France

## Abstract

The localisation of apico-basal polarity proteins along the Z-axis of epithelial cells is well understood while their distribution in the plane of the epithelium is poorly characterised. Here we provide a systematic description of the planar localisation of apico-basal polarity proteins in the *Drosophila* ommatidial epithelium. We show that the adherens junction proteins Shotgun and Armadillo, as well as the baso-lateral complexes, are bilateral, i.e. present on both sides of cell interfaces. In contrast, we report that other key adherens junction proteins, Bazooka and the myosin regulatory light chain (Spaghetti squash) are unilateral, i.e. present on one side of cell interfaces. Furthermore, we demonstrate that planar cell polarity (PCP) and not the apical determinants Crumbs and Par-6 control Bazooka unilaterality in cone cells. Altogether, our work unravels an unexpected organisation and combination of apico-basal, cytoskeletal and planar polarity proteins that is different on either side of cell-cell interfaces and unique for the different contacts of the same cell.

Each epithelium is polarised along the apico-basal axis. This polarity organises the vectorial functions of epithelia and tightly regulates the exchanges between the internal milieu and the environment. Establishment and maintenance of apico-basal polarity requires the stereotyped positioning of a highly conserved set of proteins along the Z-axis of cells (Fig. 1A)^1^,^2^,^3^.

In *Drosophila*, Crumbs (Crb) associates with Stardust (Sdt), Pals1-associated Tight Junction (Patj), Par-6, atypical protein kinase C (aPKC) (Fig. 1A) and cytoskeletal components in the most apical region of cells^1^,^2^,^4^,^5^. Immediately below the Crb domain are the proteins of the adherens junctions that mediate tissue cohesion and interfacial tension. Among *Drosophila* adherens junction proteins are Shotgun (Shg, also known as DE-cadherin), the Catenins, α (α-Cat) and β (known as Armadillo in flies: Arm) and Bazooka (Baz, also known as Par-3)^2^,^6^,^7^,^8^ (Fig. 1A). Further down, extending along the lateral membranes, are the baso-lateral proteins Discs large 1 (Dlg1), Scribbled (Scrib) and Lethal (2) giant larvae (L(2)gl) together with the *Drosophila* septate junctions proteins^2^,^9^,^10^ (Fig. 1A).

This stereotyped distribution of apico-basal polarity proteins along the Z-axis of cells is maintained by conserved regulatory loops between apical and baso-lateral polarity complexes that mutually restrict each other’s localisation while positioning and stabilising the adherens junction belt in between^1^,^2^,^7^,^8^,^9^,^10^,^11^,^12^,^13^,^14^,^15^,^16^.

In addition to apico-basal polarity, many epithelia exhibit a second polarity axis called planar polarity or tissue polarity. This polarity axis is perpendicular to apico-basal polarity, lying within the plane of the epithelium (Fig. 1B,C). Planar cell polarity (PCP) is obvious in epithelia that produce external structures such as the distally oriented hairs on the drosophila wing, the fur of vertebrates or the V-shaped stereocilia bundles found in mammalian inner ears^17^,^18^,^19^,^20^. Importantly, the planar polarity pathway is also active in tissues without external structures such as the eye disc or animal tissues undergoing convergent extension (see refs 19, 21 and 22 for reviews).

Tissue polarity is controlled by a conserved set of proteins called planar polarity proteins. The core planar polarity pathway consists of six proteins, Frizzled (Fz), Dishevelled (Dsh), Diego (Dgo), Van Gogh (Vang, also known as Strabismus), Prickle (Pk) and Starry night (Stan, also known as Flamingo)^18^,^19^,^20^,^23^,^24^,^25^,^26^,^27^,^28^,^29^,^30^. PCP proteins localise apically, overlap with adherens junctions and extend until the upper part of the baso-lateral membrane^31^. PCP proteins have the unique ability to self-organise in distinct domains in cells. In the fly wing, where planar polarity is best understood, Fz, Dsh, and Dgo localise to the distal side of cells^27^,^32^,^33^, where the hair will grow, while Vang and Pk localise to the proximal side of cells^34^,^35^ (see also Fig. 1B,C). Finally, the atypical cadherin Starry night localises to the Fz and Vang domains of the same cell^30^,^36^,^37^. Altogether, PCP proteins are unilateral, i.e. present only on one side of a cell interface (Fig. 1B,C) except for Starry night that is bilateral, i.e. present on both sides of the same interface.

The distribution of apico-basal polarity proteins along the Z-axis of cells is thought to correlate with their function and was therefore extensively studied^1^,^2^. In contrast, the distribution of apico-basal polarity proteins in the plane is largely unknown and only a handful of studies do report planar asymmetries for apico-basal polarity proteins in epithelia (**Par-1:**^38^**, Shotgun:**^39^**, Crumbs and aPKC:**^40^**, Baz:**^41^,^42^,^43^). In order to better understand the planar organisation of apico-basal polarity proteins, we decided to use a mosaic analysis strategy^44^, as done long ago for planar cell polarity. Importantly, mosaic analyses allow to determine protein uni- or bilaterality on cell interfaces, which was never addressed previously. Using mosaics, we show that apico-basal proteins organise in two distinct classes in epithelial cells. The first class, that comprises baso-lateral proteins and two adherens junction proteins, i.e. Shg and Arm, are as expected present on both sides of cell interfaces, i.e. bilateral. In contrast, the second class of apico-basal proteins, comprising Baz and the fly non-muscle myosin regulatory light chain, Sqh, are unexpectedly unilateral on epithelial interfaces. Finally, we demonstrate that the unilateral distribution of Baz is regulated by the PCP pathway but not by the apical determinants Crumbs and Par-6.

## Results

### Planar distribution of apico-basal polarity proteins in the *Drosophila* eye

**Figure 1:**
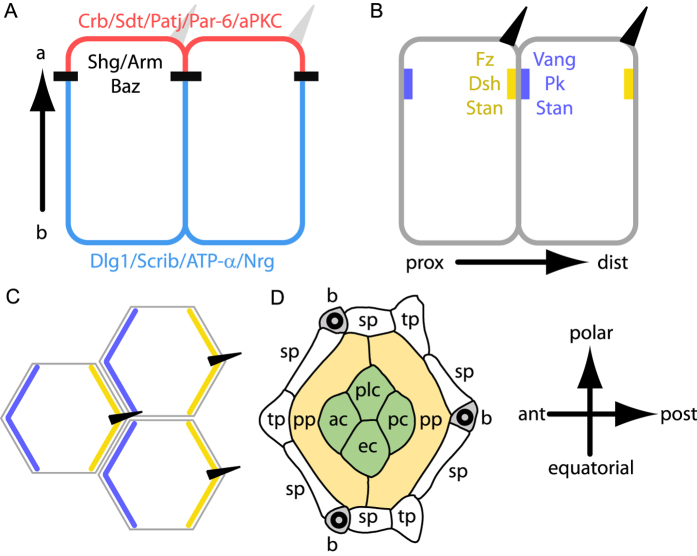
Planar and transversal distributions of apico-basal and planar polarity proteins. (**A,B**) transverse section showing the distribution of apico-basal (**A**) and planar polarity proteins (**B**) in fly wing epithelial cells. (**A**) The apical most region of the cell is shown in red, adherens junction are indicated in black and baso-lateral domains in blue. (**B**) Proximal (prox) PCP domains containing Vang, Pk and Stan are indicated in blue. Distal (dist) PCP domains containing Fz, Dsh, Dgo and Stan are indicated in yellow. Hairs (dark triangles) grow specifically from the distal side of cells. (**C**) Top view of the cells shown in (**B**). (**D**) Scheme of a 32 h APF fly ommatidium. pp, sp and tp indicate the primary, secondary and tertiary pigment cells, respectively. ac, pc, ec and plc indicate the anterior, posterior, equatorial and polar cone cells, respectively. Wild type ommatidia contain three bristles (b). Here and wherever applicable, the ommatidium is oriented with anterior left and polar up.

The fly eye epithelium is a highly structured organ composed of about 800 ommatidia. Each ommatidium contains four lens-secreting cone cells (Fig. 1D), focusing the light over eight rhodopsin-expressing photoreceptor cells^45^,^46^. The four cone cells are surrounded by two primary pigment cells, themselves in contact with several secondary and tertiary pigment cells shared between adjacent ommatidia (Fig. 1D). Each ommatidium finally contains three bristles that exhibit a chiral organisation, sufficient to determine ommatidia orientation (Fig. 1D). Altogether, these ommatidial cells organise in a highly stereotyped manner to form a hexagonal ommatidium.

To determine the planar distribution of apico-basal polarity proteins in fly ommatidia, we induced the formation of patches of clonally related cells expressing a GFP-tagged apico-basal polarity protein adjacent to patches of cells that do not express GFP (Supplementary Fig. S1). Importantly, cells lacking GFP expression are wild type; i.e. they express the endogenous protein that is not fluorescently labelled (Supplementary Fig. S1). Similarly, GFP positive cells are also functionally wild type as they express, at physiological or endogenous levels, an active protein fused to GFP (see Supplementary Fig. S1 and Methods). At the interface between GFP positive and GFP negative cells, a refined planar distribution of apico-basal polarity proteins is obtained (Supplementary Fig. S1). Such mosaics make it possible to determine the localisation of apico-basal polarity proteins with the resolution of one membrane bilayer (less than 10 nm), surpassing results that have been obtained with super resolution microscopy using STED, PALM and STORM^47^,^48^,^49^. This genetic approach allows determining unilaterality or bilaterality of proteins on contacts (Supplementary Fig. S1).

### Armadillo and Shotgun are bilateral on cell interfaces

**Figure 2:**
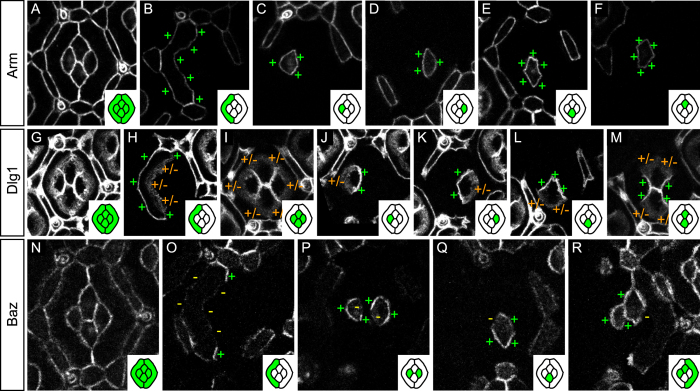
Planar distribution of apico-basal polarity proteins. (**A–F**) Arm::GFP mosaics. (**A**) Characteristic distribution of Arm::GFP in a 32 h APF ommatidial epithelium. (**B**) GFP-labeled primary pigment cell. Note the even Arm::GFP distribution around the primary pigment cell cortex (+). (**C–F**) Even distribution of Arm::GFP around the cortices of the anterior (**C**), posterior (**D**), equatorial (**E**), and polar (**F**) cone cells. (**G–M**) Dlg1::GFP mosaics. (**G**) Characteristic distribution of Dlg1::GFP in the 32 hAPF ommatidial epithelium. (**H**) Dlg1::GFP is enriched on the outer interface of the primary pigment cell (+) while its inner interface shows a diffuse Dlg1::GFP signal (+/−). (**I**) Outer cone cell interfaces show diffuse Dlg1::GFP signal (+/−) while all cone-cone interfaces (**J–M**) show a strong and sharp Dlg1::GFP signal (+). (**N–R**) Baz::GFP mosaics. (**N**) Every interface of the 32 h APF ommatidial epithelium carries Baz::GFP. (**O**) Baz distribution in primary pigment cells. Baz is devoid from outer and inner primary pigment cells interfaces (−). Baz is specifically enriched at the zone of contact between adjacent primary pigment cells (+). (**P**) In anterior cone cells (left) Baz::GFP is specifically depleted from the interface shared with the polar cone cell (−) and present elsewhere (+). In posterior cone cells (right), Baz::GFP is specifically depleted on the interface with the equatorial cone cell (−). (**Q**) In equatorial cone cells, Baz::GFP is excluded from the interface with the anterior cone cell (−). (**R**) In polar cone cells, Baz::GFP is excluded from the interface with the posterior cone cell. In this figure and the following, insets contain a cartoon representation of the ommatidia where GFP positive cells are shown in green and GFP negative cells in white. To gain space, the posterior primary pigment cell is not shown. Note however that the protein distribution in posterior primary pigment cell is mirror symmetric to that in the anterior primary pigment cell (data not shown).

In *Drosophila*, adherens junctions are composed of cadherin molecules that extracellularly mediate adhesion between cells while they intracellularly connect, via catenins to the actin cytoskeleton^2^,^50^. As adherens junctions mediate tissue cohesion, their constituent proteins are expected to localise all around the cortex. We tested this hypothesis by looking at the planar distribution of a core member of adherens junctions, the Arm protein (Fig. 2A). For simplicity, we chose to focus on the planar distribution of Arm in primary pigment and cone cells and ignored secondary and tertiary pigment cells (Fig. 1D). The Arm::GFP pattern reveals that Arm is evenly distributed around the cortex of primary pigment (Fig. 2B) and cone cells (Fig. 2C–F) and is therefore always bilateral on cell contacts.

We then looked at the distribution of Shotgun (Supplementary Fig. S2A). Shg is evenly distributed around the cortex of primary pigment cells (Supplementary Fig. S2B) similar to Arm. In cone cells, Shg is enriched on outer interfaces, in contact with the primary pigment cells (Supplementary Fig. S2C,D), while cone-cone interfaces are largely devoid of Shg (Supplementary Fig. S2A,C,D), likely due to the presence of Cadherin-N there^51^. The low amounts of Shg between cone cells prevents us from further refining the distribution of Shg on cone-cone contacts. Altogether, Shg is provided bilaterally on Shg positive contacts, similar to Arm.

### Baso-lateral proteins are present on both sides of epithelial interfaces

We then checked the planar distribution of several baso-lateral proteins. We started by looking at Dlg1 mosaics (Fig. 2G). Dlg1 is enriched on outer primary pigment cells interfaces while their inner interfaces in contact with cone cells show diffuse Dlg1::GFP signal (Fig. 2H). Cone cells exhibit the opposite pattern, i.e. they show diffuse Dlg1 signal on their outer interfaces and a strong, sharp, Dlg1 signal on their inner, cone-cone, interfaces (Fig. 2I–M). Importantly the level of expression and distribution of Dlg1 on either side of each contact is similar. In conclusion, Dlg1 is bilateral on cell interfaces; similar to Shg and Arm. All other baso-lateral and septate junctions proteins studied show a pattern strikingly similar to that of Dlg1 (compare Supplementary Fig. S2E–V with Fig. 2G–M).

### Baz is unilateral on epithelial interfaces

We then studied the localisation of Baz (Fig. 2N), an apico-basal polarity protein known to localise to adherens junctions in epithelial cells^6^,^7^,^8^. Baz::GFP mosaics reveal a surprising planar distribution that differs largely from that of Arm and Shg. In primary pigment cells, Baz is specifically enriched at the zone of contact between adjacent primary pigment cells while largely absent from other interfaces of the same cells (Fig. 2O). In cone cells, Baz is present on outer interfaces in contact with primary pigment cells (Fig. 2P–R). Furthermore, each cone cell has its own unique distribution of Baz (Fig. 2P–R). In anterior cone cells, Baz is enriched on the interface with the equatorial cone cell and depleted from the interface with the polar cone cell (Fig. 2P). The posterior cone cell shows the opposite pattern (Fig. 2P). The equatorial cone cell localises Baz on its interface with the posterior cone cell while its interface with the anterior cone cell is depleted for Baz (Fig. 2Q). The polar cone cell exhibits the opposite pattern (Fig. 2R). Finally, polar and equatorial cone cells localise Baz on both sides of their shared interface (Fig. 2Q,R). In conclusion, Baz is present on every contact of the ommatidial epithelium (Fig. 2N) but unlike Arm and Shg, it is unilateral on most interfaces.

### Baz unilaterality is not regulated by Crumbs or Par-6

We were puzzled by the novel and unexpected unilateral distribution of Baz and searched for proteins that could explain its distribution. We wondered whether the two main regulators of Baz distribution along the Z-axis of cells in *Drosophila*, Par-6 and Crumbs^7^,^8^ would account for Baz unilaterality.

**Figure 3:**
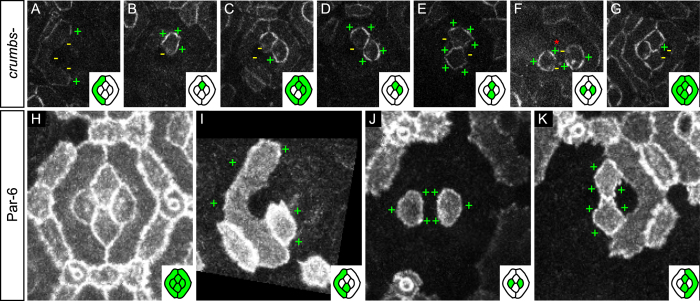
Crumbs and Par-6 do not regulate the planar distribution of Baz. (**A–G**) Baz::GFP mosaics in *crb* null mutant cells (wild type cone and pigment cells are indicated by a red asterisk). (**A,C,G**) Inner primary pigment cell interfaces are devoid of Baz signal. (**B–G**) Baz remains unilateral in *crb* null mutant cone cells. (**H–K**) Par-6::GFP mosaics. (**H**) Characteristic distribution of Par-6 in 32 h APF eyes. (**I**) Par-6 is enriched on outer (+) primary pigment cell interfaces. (**J,K**) Par-6 is evenly distributed around the cortex of cone cells (+).

We started by assessing the role of Crumbs on the planar distribution of Baz and generated dual overlapping mosaics to monitor Baz localisation in *crumbs* null mutant cells (see Methods). Loss of *crumbs* has no obvious impact on Baz planar distribution. Indeed, Baz still localises on outer interfaces of primary pigment cells and remains absent from their inner interfaces (Fig. 3A). Similarly, *crumbs* mutant cone cells still exhibit a clear unilateral Baz distribution (Fig. 3B–G).

We then wondered whether the asymmetry of Baz (Par-3) would correlate with the distribution of Par-6 since both proteins are known to localise asymmetrically to the same cell side in dividing sensory organ precursors (SOPs) and during the cleavage of the *C. elegans* egg^52^,^53^,^54^ and that Par-3 asymmetry depends on Par-6 in *C. elegans*^52^,^53^. We monitored the localisation of Par-6 using a functional Par-6 genomic rescue construct tagged with GFP (see Fig. 3H and Methods). Par-6 distribution differed largely from that of all other apico-basal protein studied, including Baz. Indeed, Par-6 is enriched on outer interfaces of primary pigment cells and lower on their inner interfaces (Fig. 3I,K). In cone cells, Par-6 is present all around the cortex (Fig. 3J,K) and therefore bilateral on cone-cone interfaces where Baz is unilateral.

Altogether, this suggests that the key regulators of Baz distribution along the Z-axis of cells, Par-6 and Crumbs, cannot account for the unilateral distribution of Baz in the 32 hours after puparium formation (h APF) ommatidial epithelium.

### The non-muscle myosin II regulatory light chain is unilateral

**Figure 4:**
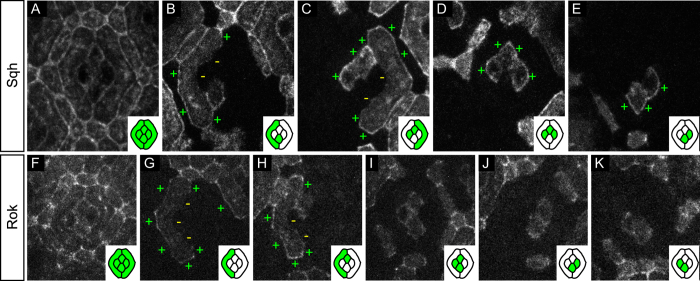
Planar distribution of Sqh and its kinase Rok. (**A–E**) Sqh::GFP mosaics. (**A**) Characteristic distribution of Sqh::GFP in the 32 h APF ommatidial epithelium. (**B**) Sqh::GFP is enriched on the outer primary pigment cell interfaces (+) and depleted from their inner interfaces (−). (**C–E**) Outer cone cell interfaces are positive for Sqh::GFP (+). All cone-cone interfaces carry low amounts of Sqh::GFP proteins, preventing us from drawing strong conclusions on the uni- or bilaterality of the protein there. (**F–K**) Rok::GFP mosaics. (**F**) Characteristic distribution of Rok::GFP in the 32 h APF ommatidial epithelium. (**G,H**) Rok::GFP is enriched on the outer primary pigment cell interfaces (+) and depleted from their inner interfaces (−). (**H–K**) Planar distribution of Rok in cone cells is highly variable, four random samples are presented here.

In fly tissues, planar localisation of Baz has been shown to negatively correlate with Myosin II distribution^41^,^42^. We therefore decided to look at the distribution of Myosin II to see if it could explain the observed Baz asymmetries. We generated mosaics expressing GFP-tagged Spaghetti squash (Sqh) (Fig. 4A–E), the fly myosin regulatory light chain^55^. In primary pigment cells, Sqh is enriched on outer interfaces and depleted from inner interfaces (Fig. 4B,C). In contrast, in cone cells, Sqh is mainly enriched on outer interfaces in contact with primary pigment cells (Fig. 4C–E). Cone-cone interfaces exhibit low levels of Sqh, preventing us from drawing conclusions on Sqh uni- or bilaterality there (Fig. 4A–E). Altogether, we can conclude that Sqh, like Baz is mainly unilateral on assessable contacts. In addition, the partial overlap between Sqh and Baz patterns neither suggests recruitment nor mutual exclusion in the ommatidial epithelium.

We therefore wondered whether Sqh localisation would correlate with that of its kinase Rok (Fig. 4F–H)^56^. Rok^57^, like Sqh, is primarily found on the outer side of primary pigment cells and absent on their inner interfaces (Fig. 4G,H). In cone cells, Rok distribution is very variable and we could not detect any obvious preferred localisation (Fig. 4H–K). Altogether, this suggests that Rok may regulate Sqh distribution in primary pigment cells, whereas other kinases are probably at work in cone cells.

### Planar distribution of core planar cell polarity proteins in the eye epithelium

As the unilateral distribution of Baz is not dependent on Crumbs and cannot be explained from the bilateral distribution of other apico-basal polarity proteins studied, including Par-6, we searched for additional unilateral proteins that could bias Baz distribution. Planar polarity proteins fulfil the unilaterality criterion in epithelia and are therefore strong candidates.

Planar polarity protein distribution is well established in numerous tissues, including the fly eye, however the latter studies have focused on the distribution of PCP proteins in neuronal cells during eye disc development^21^. Consequently, the distribution of PCP proteins in epithelial (i.e. non-neuronal) cells at late developmental stages was not known and hard to infer from planar distributions in the disc due to the high number of cell rearrangements that occur during eye development. We therefore decided to investigate it by generation of mosaics using the classical flip-out constructs that express fluorescently tagged PCP proteins^32^,^58^,^59^ (see also Methods).

### Unilateral distribution of Vang and Baz overlap

**Figure 5:**
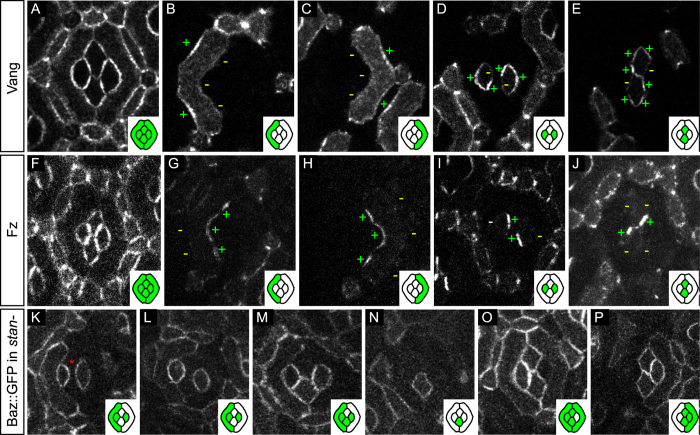
Planar organisation and function of PCP proteins in the eye. (**A–E**) Vang::YFP mosaics. (**A**) Characteristic distribution of Vang::YFP in 32 h APF ommatidia. (**B,C**) Vang::YFP is enriched on outer interfaces of primary pigment cells (+) and depleted on their inner interfaces (−). (**D,E**) Vang::YFP in cone cells. (**D**) Vang::YFP is depleted (−) in anterior cone cells (left) at their interface with polar cone cells and in posterior cone cells (right) at their interface with equatorial cone cells. (**E**) Polar cone cells (top) are devoid of Vang::YFP signal (−) at their interface with the posterior cone cells and equatorial cone cells (bottom) are devoid of Vang::YFP signal (−) at their interface with anterior cone cells. (**F–J**) Fz::YFP mosaics. (**F**) Characteristic distribution of Fz::YFP in ommatidia. (**G,H**) In primary pigment cells, Fz is enriched on inner interfaces in contact with cone cells (+) and depleted elsewhere (−). (**I,J**) Fz is enriched on one interface per cone cell. (**I**) Fz is enriched in anterior cone cells (left) at the interface with polar cone cells (+). In posterior cone cells (right) Fz is enriched on the interface with equatorial cone cells (+). (**J**) Fz is enriched in polar cone cells (top) at the interface with posterior cone cells (+). In equatorial cone cells (bottom) Fz is loaded at the interface with anterior cone cells (+). Altogether, the Fz pattern is the negative of the Vang pattern. (**K–P**) Baz::GFP mosaics in *stan* null mutant cells (remaining wild type cells are indicated by asterisks). (**K–P**) Improper ommatidial rotation^60^,^89^ and misplaced bristles in *stan* mutants prevent us from determining the antero-posterior and the polar-equatorial axes. Therefore, ommatidia are oriented using the long axis of primary pigment cells in (**K–P**). (**K–M**) Polar/equatorial cells express Baz::GFP on their contacts with anterior/posterior cone cells, i.e. no Baz::GFP depletion is observed (compare with Fig. 2P). Baz::GFP shows an even distribution around the cortex of isolated anterior/posterior (**L**) and polar/equatorial (**N**) cone cells. (**O,P**) Polar/equatorial cells express Baz::GFP on their shared interface with anterior/posterior cone cells (compare with Fig. 2Q,R).

We first monitored the planar distribution of Vang (Fig. 5A), a marker of the proximal side of cells in the fly wing (Fig. 1B,C). In the 32 h APF ommatidial epithelium, Vang intensity in primary pigment cells peaks at the center of the zone of contact with the secondary pigment cells and is lower around the vertices of these contacts (Fig. 5B,C). Vang is largely depleted form inner primary pigment cell interfaces (Fig. 5B,C). Strikingly, in cone cells, the distribution of Vang is reminiscent of that of Baz (compare Fig. 5D,E to Fig. 2P–R). Note that due to weak PCP signal in photoreceptor cells at pupal stages, we could not address PCP asymmetries in mature photoreceptor cells.

Altogether, the distribution of Vang in cone cells overlaps with that of Baz but is poorly correlated in primary pigment cells, suggesting that the PCP pathway may control Baz planar distribution in cone cells but not in primary pigment cells.

### Fz distribution is opposite to that of Vang

We then investigated the distribution of Fz, a marker of the distal side of the cell in the wing epithelium (Fig. 1B,C). Similar to results in other tissues, the Fz pattern is complementary to that of Vang. Fz is depleted from outer interfaces of primary pigment cells and enriched on inner interfaces with a peak on the central region of the contacts (Fig. 5G,H). In cone cells, Fz is absent on outer interfaces (Fig. 5I,J). In between cone cells, Fz is present on the side of contacts that is depleted for Vang, i.e. Fz is present in anterior cone cell on the interface with the polar cone cell (see left cell in Fig. 5I) and in posterior cone cells on the interface with equatorial cone cell (see right cell in Fig. 5I). Similarly, Fz is present in equatorial cone cell at the interface with the anterior cone cell (see lower cell in Fig. 5J) and in polar cone cells at the interface with the posterior cone cell (see upper cell in Fig. 5J).

Altogether, the distribution of Fz is opposite to that of Vang and Baz in cone cells.

### The PCP pathway controls the unilateral distribution of Baz in cone cells but not Dlg1 asymmetries

The strong correlation between the distributions of Vang and Baz in cone cells suggests that PCP may regulate Baz unilaterality. To test this hypothesis we monitored Baz distribution in *stan* mutant cells (see Methods); Stan is a PCP protein required for the recruitment of Fz and Vang to their respective distal and proximal domains^32^,^34^. Importantly, since *stan* affects the orientation of ommatidia^60^ as well as the number and position of bristles we use as landmarks, we are unable to precisely orient ommatidia in these mutants. We therefore chose to align ommatidia using the long axis of primary pigment cells. Using this axis, we can differentiate polar and equatorial cone cells from the anterior and posterior ones, but we can neither distinguish the polar from the equatorial cone cell nor the anterior from the posterior cone cell. In any case, Baz appears evenly distributed around the cortex of *stan* mutant cone cells (Fig. 5K–P). This observation contrasts with the control situation where cone cells always have one contact depleted for Baz (Fig. 2P–R and compare to Fig. 5K–P). Altogether, this indicates that Baz becomes bilateral on cone-cone interfaces in PCP mutants and that PCP acts as a switch between unilateral and bilateral distributions of Baz. We also note that Baz distribution is maintained in primary pigment cells mutant for *stan* (Fig. 5K–M, also compare with Fig. 2O), suggesting that Baz localisation in these cells is PCP-independent.

Since several studies in flies and vertebrates report physical and genetic interactions between the PCP protein Vang and the baso-lateral proteins, Dlg1 and Scrib^61^,^62^,^63^,^64^,^65^, we wondered whether PCP would also regulate the asymmetric distribution of baso-lateral proteins in the eye. We again performed dual mosaics to follow the planar distribution of Dlg1::GFP in cells mutant for *stan* (see Supplementary Fig. S3 and Methods). However, Dlg1 distribution remained unchanged in *stan* mutant cells (compare Supplementary Fig. S3A–F to Fig. 2H–M).

Altogether, this suggests that the PCP pathway specifically controls the planar distribution of the adherens junction protein Baz but not that of baso-lateral proteins.

## Discussion

**Figure 6:**
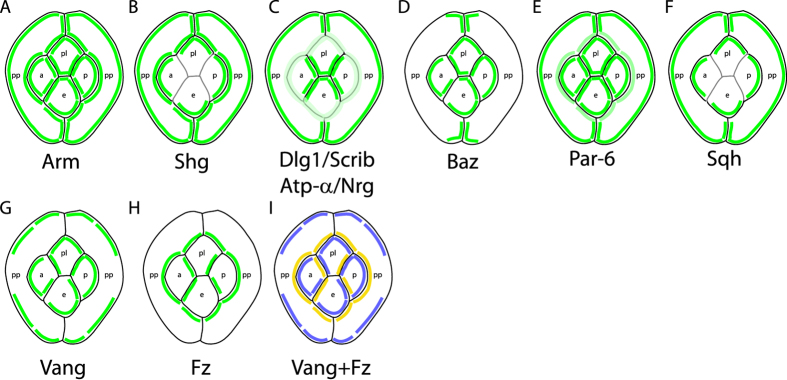
Schematic representation of the planar distribution of apico-basal and planar polarity proteins in the ommatidial epithelium. (**A–I**) Planar distribution of (**A**) Arm, (**B**) Shg, (**C**) Dlg1/Scrib/ATP-α/Nrg, (**D**) Baz, (**E**) Par-6, (**F**) Sqh, (**G**) Vang and (**H**) Fz. (**I**) Combined planar distribution of Fz (yellow) and Vang (blue). Note the complementary distributions of Fz and Vang proteins. Due to the weakness of the signal on the interfaces between cone cells, Shg (**B**) and Sqh (**F**) planar localisation is not represented in (**B,F**). Similarly the weak PCP signal for Fz and Vang on the interface between equatorial and polar cone cells prevents us from drawing strong conclusions on the planar distribution of PCP proteins on this interface.

The distribution of apico-basal polarity proteins along the Z-axis of epithelial cells has been the subject of intense research while their distribution in the plane of the epithelium is largely unknown. Here we provide the first systematic study of the planar distribution of apico-basal polarity proteins in the fly eye (Fig. 6). We find that Baz and Sqh are enriched on one side of epithelial cell interfaces, i.e. are unilateral (Fig. 6D,F) whereas Arm, Shg and baso-lateral proteins are present in similar quantities on either side of cell contacts, i.e. are bilateral (Fig. 6A–C). In addition, we demonstrate that the planar distribution of Baz is controlled by planar cell polarity in cone cells.

### Interplay between apico-basal polarity and planar cell polarity

Links between PCP and apico-basal polarity have been previously reported in dividing sensory organ precursors in the fly notum (SOPs)^61^,^66^,^67^,^68^,^69^. There, Fz localises the Baz/Par-6/aPKC complex to the posterior side of cells while Vang recruits Dlg1 to the anterior side of the same cell. In contrast, in the eye, Baz localises with Vang rather than with Fz (this study). Another striking difference is that Baz unilaterality and asymmetry in the 32 h APF eye requires PCP signaling (this study), whereas Baz/Par-6/aPKC asymmetries can still form in dividing SOPs of PCP mutant animals^66^,^67^. Even if the latter result may suggest, at first glance, that PCP proteins are not required for asymmetric segregation of apico-basal proteins in SOPs, a recent study^70^ proposed that in the presence of PCP, Baz/Par-6/aPKC asymmetric localisation establish several hours before SOP division. In PCP mutants, the same authors report that Baz/Par-6/aPKC asymmetries are not detected prior to division^70^. Altogether, these results suggest that PCP may function similarly in SOPs and in the eye epithelium by promoting Baz asymmetries. However, in the case of the 32h APF eye epithelium, Par-6 does not localise with Baz; this result is consistent with the fact that Baz and Par-6 are present in different Z regions of epithelial cells^7^,^8^,^71^,^72^. Interestingly, Besson *et al*. report that Baz is always symmetrically localised in notum epithelial cells and hence should be bilateral at adherens junctions. This result contrasts with our observation that Baz is unilateral in ommatidial cells and suggests notum and eye epithelia may be different. Importantly, some protein asymmetries may only be detected using mosaics due to the limited resolution of microscopes (compare Fig. 2N to Fig. 2O–R and Fig. 4A to Fig. 4B–E). It may therefore be interesting to revisit Baz asymmetries in the notum and other fly epithelia using mosaics.

In the eye imaginal disc, the precursor of the pupal eye, apico-basal polarity proteins have been proposed to regulate PCP signaling by controlling Fz phosphorylation^31^. From this work, apico-basal polarity proteins act upstream of PCP. In contrast, our study unravels a novel regulation of apico-basal polarity proteins downstream of PCP. In addition, we observe, a strong colocalisation between Vang and Baz that calls for an interaction, direct or indirect, between Baz and Vang. Direct binding between those two proteins could, however, not detected using yeast two hybrid assays^31^,^65^, suggesting the interaction might be indirect. In an alternative scenario, Djiane *et al*. report a direct but weak binding between Baz and Fz that could for example target Baz for degradation or endocytosis and lead to a specific depletion of Baz on Fz positive contacts. Further biochemical experiments are required to discriminate between those different Baz sorting mechanisms.

Finally, a third link between Baz and PCP was proposed^73^. There, the authors describe that PCP is perturbed upon strong overexpression of Baz and show that Baz can bind specifically to the PDZ binding domain of the so-called Stan isoform of Stan. However, the physiological relevance of this binding remains unclear^73^. In the case of the 32 h APF eye ommatidium, we think this interaction is unlikely since Stan is known to be a bilateral protein and should therefore recruit Baz bilaterally on cone cell interfaces, which is not what we observe.

### Each epithelial contact is characterised by a unique apico-basal polarity signature

In conclusion, we report a surprising feature of epithelial cells, i.e. different contacts of a given cell have different amounts and distributions of apico-basal, planar polarity and cytoskeleton proteins, suggesting that the cortical distribution of proteins is not uniform in epithelial cells. In contrast, each cell-cell interface defines its own apico-basal, planar and cytoskeleton protein signature independently of the other interfaces of the same cell.

How this contact specific distribution is established remains to be determined. It is likely that mechanisms known to bias the distribution of planar polarity proteins, such as the oriented microtubule web^38^,^74^ could explain how two opposite cell contacts acquire different signatures, but additional, yet uncharacterised mechanisms must be at work on the remaining contacts of the same cell. In addition, it is also hard to reconcile these contact specific signatures and protein unilaterality with the bilateral vision of the establishment and maintenance of apico-basal polarity. Altogether, our observations therefore call for a re-evaluation of apico-basal proteins interactions in epithelial cells. In particular, a very challenging but exciting task will be to discriminate between the planar and the apico-basal specific functions of apico-basal polarity proteins. No doubt that *Drosophila* genetic screening capabilities will be useful to identify sets of genes that do affect the planar distribution of apico-basal polarity proteins and cytoskeleton components while keeping their Z distribution unchanged.

Finally, it will be important to study the function of the asymmetries described here. Since Baz and Sqh modulate cell adhesion and contractility, respectively^39^,^43^,^49^,^75^, they can together regulate tension at cell interfaces and hence control cell shape and morphogenesis. In addition, because myosin is mainly provided by one cell at a shared interface, the lengthening or shrinking of this interface is under the decision of a single cell. Thereby this cell has a unique role in shaping the eye that cannot be compensated by its neighbour; this may explain how cells with different fates can have specific functions during morphogenesis. Altogether, measuring the mechanical properties of eye cell interfaces, correlating them with the planar distribution of proteins and designing a physical model of the ommatidium will allow to address the roles of protein unilateralities during eye morphogenesis.

## Methods

### Fly genetics and heat shock treatments

High resolution planar distribution of apico-basal polarity proteins was inferred from the presence or absence of GFP signal at the boundaries of flippase induced mitotic clones (refs 76 and 77, see also Supplementary Fig. S1). To study the planar distribution of apico-basal polarity proteins we recombined GFP-tagged apico-basal proteins with FRT sites^76^,^77^. Hereafter is a list and description of all the flies used in this sudy. *FRT19A baz::GFP* (constructed upon the FlyTRAP^78^,^79^,^80^ line CC01941), *FRT42D shg::GFP* (constructed upon the s*hotgun* knock-in line^81^), *FRT19A arm::GFP* (constructed upon the *arm* knock-in line^82^, a gift from the P.F. Lenne team) and *FRT40A sqh::GFP* (constructed upon bloomington stock #57144) were used to monitor the distribution of Bazooka, Shotgun, Armadillo and Spaghetti Squash (the fly myosin regulatory light chain) at adherens junctions, respectively. *FRT19A dlg1::GFP* (constructed upon FlyTRAP line YC0005) and *FRT82B scrib::GFP* (constructed upon FlyTRAP line CA07683) were used to study the planar distribution Discs Large and Scribbled on baso-lateral interfaces. *FRT19A nrg::GFP* (constructed upon FlyTRAP line G00305) and *FRT82B ATP*α*::GFP* (constructed upon FlyTRAP line YC0031) were used to study the planar distribution of Neuroglian and the Na+/K+ exchange pump at septate junctions, respectively. *FRT9-2, par-6*^Δ*226*^*, par-6::GFP* flies (refs 70 and 83, a gift from F. Schweisguth) were used to study the planar distribution of Par-6, an apical protein, in the eye. Note that the Par-6::GFP construct is a functional genomic rescue construct for Par-6 that saves the *par-6*^Δ*226*^ lethal allele present on the same chromosome. Eye mosaics were generated using the ey-Flp system (initially developed by B. Disckson but directly obtained from the Bloomington stock center). *sqh::GFP* and *rok::GFP* (from Bloomington stock center) are genomic constructs driven by the *sqh* promoter that mildly overexpress these fusion proteins in cells. Note that Sqh::GFP is known to rescue all defects of the *sqh* null mutant. All other (Fly)TRAPs and knock-ins used in this study are expressed at endogenous levels from their own promoter, in their endogenous locus and are viable even when they are the only source of protein in the animal, indicating that they are fully functional GFP-tagged proteins. *FRT19A/FRT19A baz::GFP; FRT42D GMR myr-RFP/FRT42D stan*^*E59*^*; eyFlp/*+ *and FRT19A/FRT19A dlg1::GFP; FRT42D GMR myr-RFP/FRT42D stan*^*E59*^*; eyFlp/*+ were used to generate independent mosaics where a subset of *stan* null mutant cells (labeled by the absence of RFP) express Baz::GFP or Dlg1::GFP, respectively. *FRT42D stan*^*E59*^ is Bloomington stock #41776. *FRT19A/FRT19A baz::GFP; eyFlp/*+*; FRT82B ubi-RFPnls/FRT82B crb*^*11A22*^ were used to generate dual mosaics where a subset of *crb* null mutant cells express Baz::GFP. *FRT82B crumbs*^*11A22*^ is a gift from E. Knust^84^,^85^. *actin5C*>*stop*>*Vang::YFP* and *actin5C*>*stop*>*fz::YFP* constructs^32^,^58^,^59^ were used to follow the planar distribution of the Fz and Vang PCP proteins in the 32 h APF ommatidial epithelium, respectively. PCP-YFP mosaics were induced by heat shocking white pupa (0 h APF) for 5 to 15 minutes at 37 °C. In all cases, flies were collected at white pupa stage (0 h APF) and kept at 25 °C until dissection and imaging at indicated times.

### Imaging

Pupae were prepared for imaging as previously described^86^ except that eyes instead of wings were imaged. Images were acquired using 63X oil immersion objective on a Zeiss LSM 510 system equipped with classical PMTs or a Leica SP8 microscope equipped with PMTs and hybrid detectors.

### Figures

Adherens junction protein images used in this study are either single optical sections or Z projections of a few Z planes around the brightest apical focal plane. For baso-lateral and septate junction images, we used single sections or maximum projections of the most apical part of baso-lateral junctions. Presented ommatidia are representative examples collected from typically 3–10 independent eyes (each containing several hundreds independent ommatidia). In all cases, photoreceptors located below the cone cells were excluded from projections. Z projections were created using FIJI^87^. Figures were mounted using ScientiFig^88^ and Illustrator (Adobe).

## Additional Information

**How to cite this article**: Aigouy, B. and Le Bivic, A. The PCP pathway regulates Baz planar distribution in epithelial cells. *Sci. Rep.* **6**, 33420; doi: 10.1038/srep33420 (2016).

## Supplementary Material

Supplementary Information
