# Supplementary material for: The PCP pathway regulates Baz planar distribution in epithelial cells
Source: Sci Rep. 2016 Sep 14;6:33420. doi: 10.1038/srep33420 (PMC5022056; doi:10.1038/srep33420)
Supplement: Supplementary Information [file srep33420-s1.pdf]

# The PCP pathway regulates Baz planar distribution in epithelial cells

Benoit Aigouy[1] and André Le Bivic[1]

## Supplemental Information

**Figure S1 | Visualization of membrane-associated protein distributions using mosaics**

(A) Heterozygous cell encoding for GFP-tagged proteins (blue chromosome) and endogenous (unlabeled) proteins (red chromosome).

(B) Chromosome duplication.

(C, D) Flippase-induced mitotic recombination (triangles indicate recombination sites).

(E, E’, E”) Possible chromosome arrangements at metaphase.

(F) Progeny of (E). Both daughters express GFP-tagged proteins (green).

(F’) Progeny of (E’). The left and right daughter cell express GFP-tagged (green) and unlabeled proteins (gray), respectively.

(F”) Progeny of (E”). The right and left daughter cell express GFP-tagged (green) and unlabeled proteins (gray), respectively.

(G-I) Bilateral proteins at interfaces (case I).

(G) The two daughters (generated in F) put GFP-tagged proteins on their shared interface (green lines).

(G’) The shared interface appears GFP+ using confocal microscopy.

(H) The A and B daughter cells load their shared interface with GFP-tagged (green line) and unlabeled proteins (gray line), respectively.

(H’) The shared interface appears GFP+ using confocal microscopy.

(I) The B and A daughter cells load their shared interface with GFP-tagged (green line) and unlabeled proteins (gray line), respectively.

(I’) The shared interface appears GFP+ using confocal microscopy. The presence of GFP on the shared interface is independent of the position of the GFP+ cell (compare H and I). This behavior indicates the protein is bilateral on the interface.

(J-L) Unilateral protein distribution at interfaces (case II).

(J) The two daughter cells (generated in F) express GFP-tagged proteins but only the A cell puts GFP-tagged proteins on the common interface (green line).

(J’) The shared interface appears GFP+ using confocal microscopy.

(K) The A cell loads the interface shared with the B cell with GFP-tagged proteins (green line), the B cell doesn’t put proteins there.

(K’) The shared interface appears GFP+ using confocal microscopy.

(L) The A cell loads the interface shared with the B cell with unlabeled proteins (gray line), the B cell doesn’t put proteins there.

(L’) The shared interface appears GFP- using confocal microscopy. The presence of GFP signal on the shared interface depends on the position of the GFP+ cell (compare K and L). This behavior indicates protein unilaterality.

**Figure S2 | Planar distribution of Shg, Scrib, Nrg, ATP-α and Par-6 in the 32h APF eye ommatidial epithelium**

(A-D) Shg::GFP mosaics. (A) Characteristic distribution of Shotgun in 32hAPF eyes. Shg is depleted from Cadherin-N rich cone-cone interfaces (-). (B) Shg::GFP is evenly distributed around the primary pigment cell cortex. (C, D) GFP+ cone cells. Note the Shg enrichment on outer cone cell interfaces (+).

(E-V) Planar distribution of GFP tagged baso-lateral proteins. (E-J) Scrib::GFP mosaics. (K-P) Nrg::GFP mosaics. (Q-V) ATP-α::GFP mosaics. The distribution of baso-lateral proteins is

reminiscent of that of Dlg1 **(compare Supplementary Fig. S2 E-V to Fig. 2 Q-W)**. Briefly, baso-lateral proteins are strongly enriched on outer primary pigment cell interfaces while their inner interfaces show diffuse signal (+/-) (F, L, R). Outer cone cell interfaces show diffuse baso-lateral signal (+/-) while their inner interfaces are strongly labelled with baso-lateral proteins (+) (G-J, M-P, S-V).

**Figure S3 | Planar distribution of Dlg1 is PCP-independent**

(A-F) Dlg1::GFP mosaics in *stan* null mutant cells. *stan* mutant ommatidia are oriented using the method described in **Fig. 5**. (A-C) isolated primary pigment cells show sharp Dlg1 signal on their outer and diffuse Dlg1 signal on their inner interfaces (same as wild-type, compare to **Fig. 2H**). (A-F) Polar/equatorial and antero/posterior cone cells show diffuse Dlg1 signal on their outer interfaces and strong sharp Dlg1 signal on cone-cone interfaces (same as wild-type, compare to **Fig. 2I-M**).

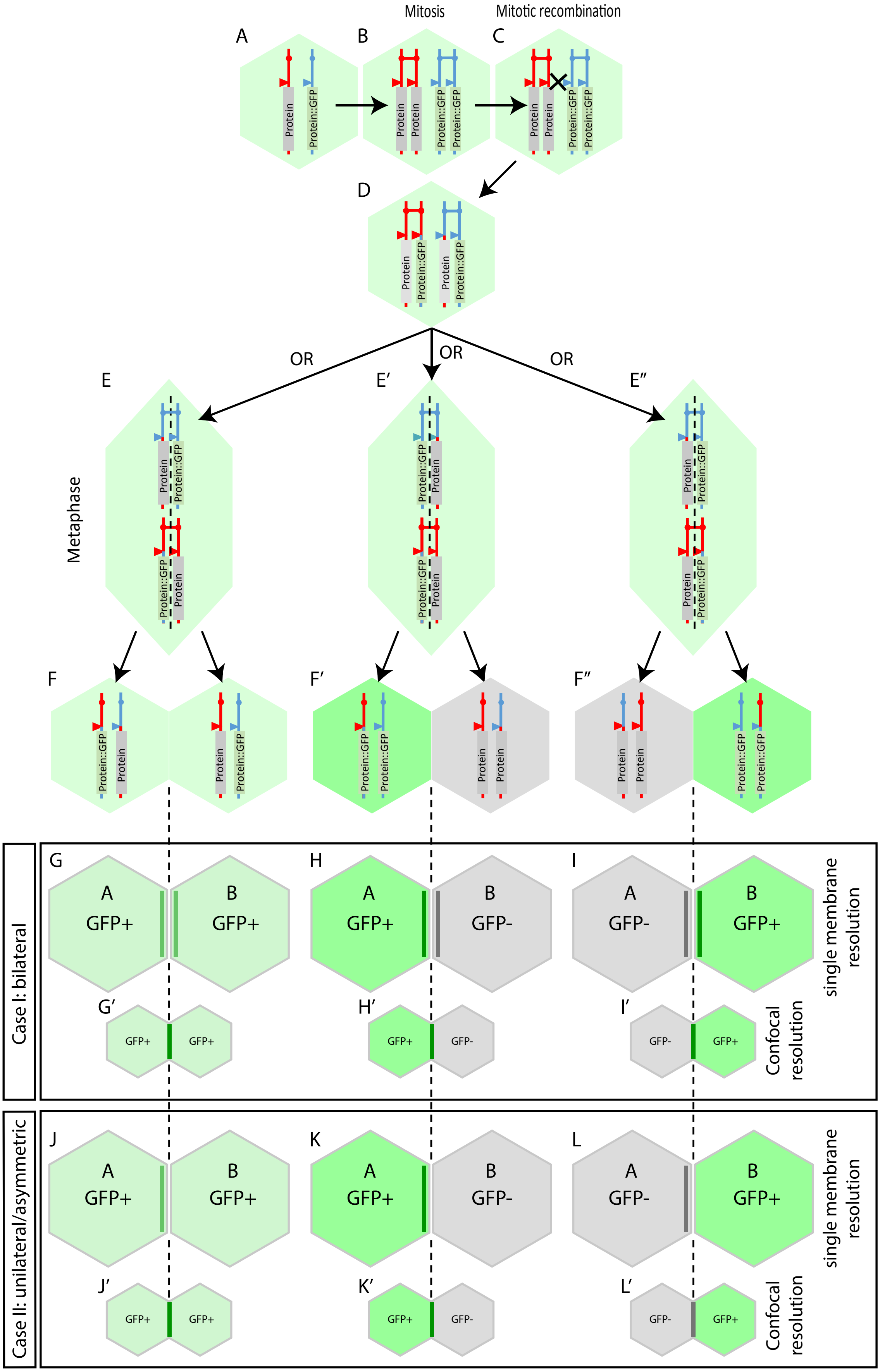


Supplementary Figure S1

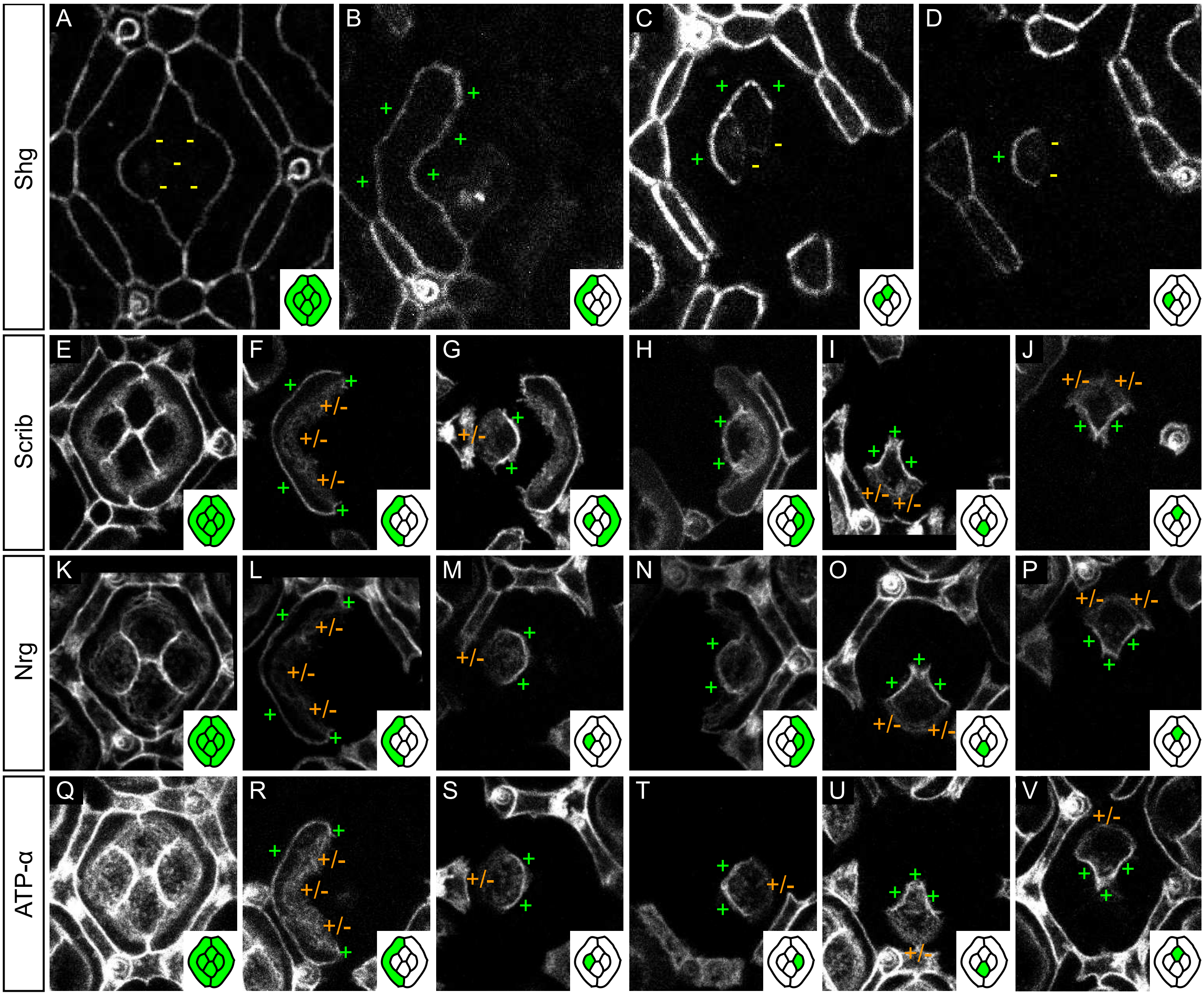


Supplementary Figure S2

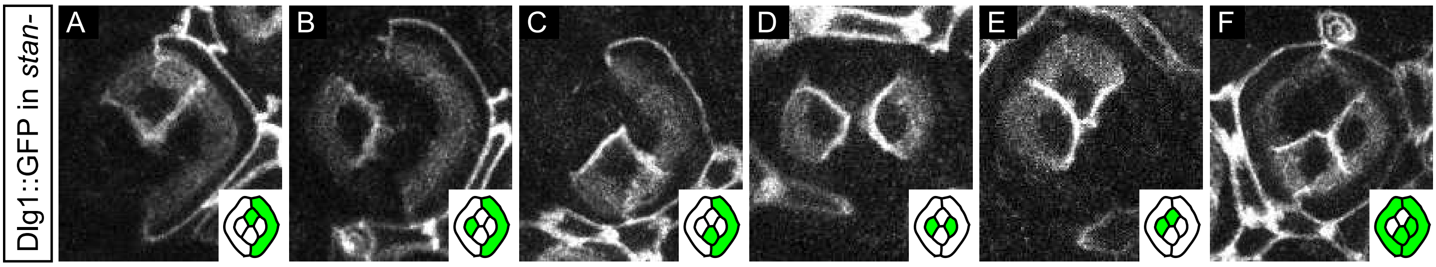


Supplementary Figure S3
